# Small cell carcinoma of the kidney: a case report and analysis of data from the Surveillance, Epidemiology, and End Results registry

**DOI:** 10.1186/s13256-018-1965-8

**Published:** 2019-03-07

**Authors:** Charles Chu, Chung-Yuan Hu, Rashmi Batra, Albert Y. Lin

**Affiliations:** 1 Department of Medicine, Santa Clara Valley Medical Center, 751 S Bascom Ave, San Jose, CA 95128 USA; 2 MD Anderson Cancer Center, Houston, TX USA; 3 Department of Pathology, Santa Clara Valley Medical Center, San Jose, California USA; 4 Department of Medicine, Palo Alto VA, Palo Alto, California USA; 5 Department of Medicine, Stanford University School of Medicine, Stanford, California USA

**Keywords:** Small cell cancer, Small cell cancer of the kidney, SEER database, Syndrome of inappropriate antidiuretic hormone secretion

## Abstract

**Background:**

Primary small cell carcinoma of the kidney is an extremely rare neoplasm. The clinical features of small cell carcinoma of the kidney are not well established due to its rarity and scarcity of case reports. We present an unusual case of small cell carcinoma of the kidney complicated by syndrome of inappropriate antidiuretic hormone secretion. We identify cases using a population-based dataset from the Surveillance, Epidemiology, and End Results registry and compare small cell carcinoma of the kidney with small cell carcinoma of the lung.

**Case presentation:**

A 69-year-old Filipino man presented with hematuria for 1 month. A computed tomography scan demonstrated a large left kidney mass with biopsy demonstrating small cell carcinoma. Within 2 months he developed dizziness and was found to have a metastatic lesion to his brain. He was hyponatremic due to syndrome of inappropriate antidiuretic hormone secretion. He did not receive chemotherapy due to his poor functional status. He died within 8 months of presentation.

**Results:**

From 1973 to 2013, 60 cases with small cell carcinoma of the kidney were identified in the Surveillance, Epidemiology, and End Results registry. Most (62%) presented with extensive stage, which occurred predominantly in white men in their seventh decade. The median overall survival with extensive stage small cell carcinoma of the kidney was 3 months versus 11 months with limited stage of small cell carcinoma of the kidney; this was worse than small cell carcinoma of the lung with a median survival of 5 and 13 months, respectively.

**Conclusion:**

We present a rare case of small cell carcinoma of the kidney complicated by syndrome of inappropriate antidiuretic hormone secretion. This adds to our understanding of the clinical features of small cell carcinoma of the kidney. Furthermore, this is the first population-based study of small cell carcinoma of the kidney using the Surveillance, Epidemiology, and End Results database. Analysis shows that overall survival is worse in small cell carcinoma of the kidney relative to that of small cell carcinoma of the lung. Small cell carcinoma of the kidney presents very aggressively, and further studies are needed to develop a standard of care.

## Background

Small cell carcinoma of the kidney (SCCK) is a rare malignancy. Extrapulmonary small cell carcinoma (SCC) is increasingly identified as an entity distinct from primary small cell carcinoma of the lung (SCCL). SCC of the urogenital tract occurs most often in the bladder. In a large reported series of 120 patients with extrapulmonary small cell cancer, only 19% of cases had primary tumors in the genitourinary tract, none of which were in the kidney [1]. Due to its rarity, the clinical course of SCCK is not well understood. Furthermore, no clear guidelines or standard of care have been established [2]. A review of the literature demonstrated few case reports that have been published thus far [2–4]. One of these studies looked at 22 cases between 1966 and 2002 and showed a median survival of 8 months [2]. However, another study estimated a mean survival of 15 months among 13 cases [3]. The lack of cases makes it difficult to properly characterize its clinical features.

Our aim in this case report is to present an unusual feature of SCCK and to understand SCCK in a larger epidemiologic context using the Surveillance, Epidemiology, and End Results (SEER) registry. We report a case of primary SCCK with metastasis to the brain complicated by syndrome of inappropriate antidiuretic hormone secretion (SIADH) who did not receive chemotherapy. We also present an analysis of a population-based cancer database, the SEER registry, which covers approximately 28% of the US population; the SEER registry serves as a reliable resource for cancer statistics and can be used to compare the epidemiology of SCCK versus its lung counterpart. Because of the vast amount of data collected on a large number of cancers, the SEER database offers a unique opportunity to study an unusual malignancy. To the best of our knowledge, this is the first SEER-based study involving SCCK.

## Case presentation

**Fig. 1:**
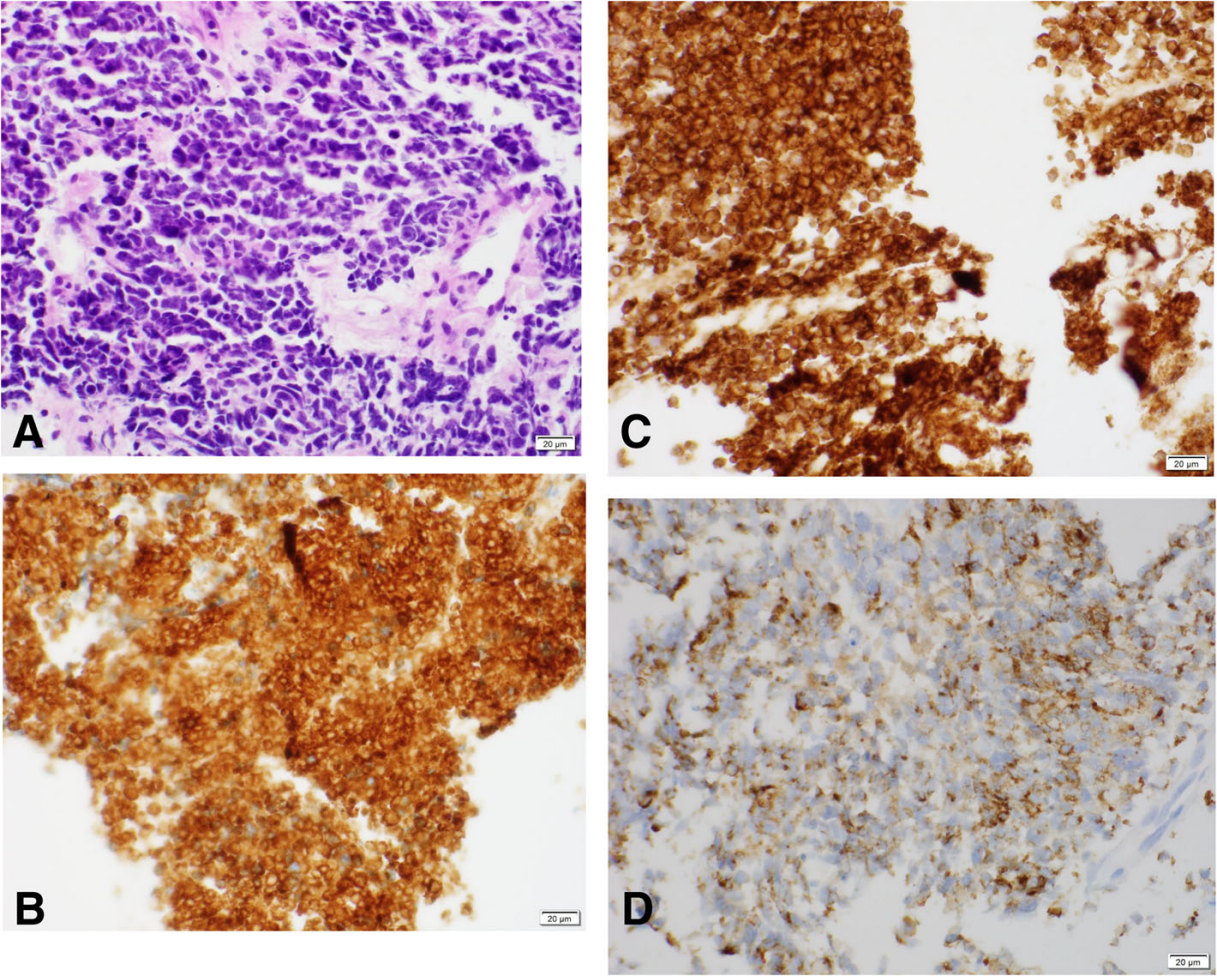
**a** Cellular neoplasm with sheets of pleomorphic round cells with hyperchromatic nuclei; × 40, hematoxylin and eosin. **b** Positive staining for neuroendocrine marker synaptophysin in the tumor cells; × 40. **c** Neuroendocrine CD56 positivity in the tumor cells; × 40. **d** Immunohistochemical findings; × 40. Dot-like cytokeratin (AE1/AE3) stain of tumor cells

A 69-year-old Filipino man with history significant for hypertension and hyperlipidemia presented to his primary care physician with hematuria with weight loss of 1 month’s duration. He did not have any flank pain, burning on urination, or increased urinary frequency. He did not endorse any symptoms of fatigue or night sweats. His only medication was atenolol for his hypertension. He did not smoke tobacco, drink alcohol, or do any recreational drugs. He was unemployed at time of interview. He did not have any family history of cancer. His vital signs were within normal limits. On physical examination, he was well appearing and in no acute distress. He had no palpable mass and had an otherwise normal cardiovascular, respiratory, and neurologic examination. Laboratory work showed normal cell counts and normal electrolytes; the results of his kidney and liver function tests were normal. A computed tomography (CT) – intravenous pyelogram was performed as a diagnostic work-up for his hematuria, which demonstrated a large mass in the left collecting system and proximal ureter. He was seen by urology with plans for surgical resection 1 month later. Three weeks later he was admitted to the Emergency Department with nausea and vomiting. He was tachycardic to 110 beats per minute but maintained a normal blood pressure. His laboratory results were notable for hemoglobin to 12.1. His sodium was 134. At that time, a CT scan of his abdomen and pelvis showed interval enlargement of the left renal mass. An ureteroscopy with biopsy was performed, which showed necrotic tissue with rare crushed degenerating atypical cells. A screening chest CT scan was also obtained which showed a small 3 mm nodule in the lower lobe of his left lung. A follow-up interventional radiology-guided left kidney biopsy showed a cellular neoplasm with sheets of pleomorphic round cells with hyperchromatic nuclei, irregular nuclear outlines, and inconspicuous nucleoli with scant and delicate cytoplasm which is consistent with SCC. The tumor cells were positive for the neuroendocrine markers synaptophysin and CD56 with focal staining for chromogranin and dot-like positive staining for cytokeratin (AE1/AE3), supporting the diagnosis of SCC (Fig. 1). A bone scan did not show any metastatic lesions. Shortly afterwards, he developed dizziness and an MRI of his brain was obtained revealing a 1.6 cm partially hemorrhagic round mass with surrounding edema in the midline superior vermis potentially representing metastatic disease. An additional 4–5 mm hemorrhagic metastatic focus was seen in the right occipital convexity. The cerebellar mass was resected and probably represented a renal origin due to the absence of lung masses along with clinical and radiographic correlation. He was started on whole brain radiation therapy during his in-patient stay. An out-patient oncology referral was made but he was unable to establish care due to frequent hospitalizations. He had several hospital admissions for nausea and vomiting and continued to decline functionally. He developed chronic hyponatremia during these hospitalizations which were attributed to SIADH. He originally presented with sodium of 119 and was stabilized to a sodium level of 128 with the use of salt tablets. He declined chemotherapy when it was offered by the oncology team during in-patient consultation due to poor quality of life and functional status; he died within 8 months of presentation at his nursing facility. The cause of his death was unknown. An autopsy was not performed.

## Materials and methods

### Study population

Data were derived from the SEER of the US National Cancer Institute: a population-based cancer database, covering more than 25% of US population [5]. Using Case Listing session of SEER*Stat (version 8.3.4), we queried cases from SEER 18 Registries 1973–2013 dataset (November 2015 submission), containing cases diagnosed within the following geographic areas: the Atlanta, Connecticut, Detroit, Hawaii, Iowa, New Mexico, San Francisco-Oakland, Seattle-Puget Sound, Utah, Los Angeles, San Jose and Monterey, Rural Georgia, the Alaska Native Tumor Registry, Greater California, Kentucky, Louisiana, New Jersey, and Greater Georgia. Due to the impact Hurricane Katrina had on Louisiana’s population from July to December 2005, SEER excluded Louisiana patients diagnosed for that time interval.

The inclusion criteria for the analysis were as follows: (1) all patients diagnosed as having SCC by the corresponding International Classification of Diseases for Oncology (ICD-O) codes of 8041–8045; (2) microscopically confirmed malignancy; and (3) one primary cancer only or the first of second or more primaries [6]. Cases were excluded in the analysis if their age at diagnosis was unknown or cause of death and follow-up time was not available or unknown. We obtained variables for each case including patient demographics (race/ethnicity, sex, age), type of radiation administered, and survival length. Their stage was considered as “limited” if they had localized or regional disease per SEER database, and extensive if they had distant disease per SEER database.

### Statistical analysis

We used chi-squared tests to compare the demographic and clinical characteristics (age, gender, race, and use of radiation) of cases of SCCK versus cases of SCCL. Overall survival (OS) was calculated from the date of diagnosis to the date of death and was analyzed using the Kaplan–Meier method. The log-rank test was used to compare the survival curves. All statistical analyses and curves of survival probabilities were conducted by using SPSS software, version 25 (SPSS Inc., Chicago, IL, USA). All reported *p* values are two-sided, and an alpha level of 0.05 was considered to indicate statistical significance.

## Results

During 1973–2013, 98,457 cases of SCC, either pulmonary or of extrapulmonary origin, were identified in the SEER registry. Of these cases, 95.7% or 94,238 were SCCL (Table 1). In contrast, only 60 cases of SCCK, accounting for 0.06% all SCC cases, were identified during the same period. More than half, or 62%, of patients with SCCK presented with extensive disease. In contrast, extensive disease was noted in 70% of patients with SCCL at diagnosis. The median age of diagnosis for SCCL and SCCK was 67 and 70 years, respectively. Male gender accounted for 65% of cases of SCCK, versus 50% in SCCL. Both SCCL and SCCK were consistently higher in whites compared with other racial groups, accounting for more than 80% of population in either condition. Less than 20% of cases of SCCK, either limited or extensive disease, received radiation therapy. In contrast, more than 50% of patients with SCCL with limited disease and two-thirds of patients with SCCL with extensive disease received radiation therapy.

**Table 1:**
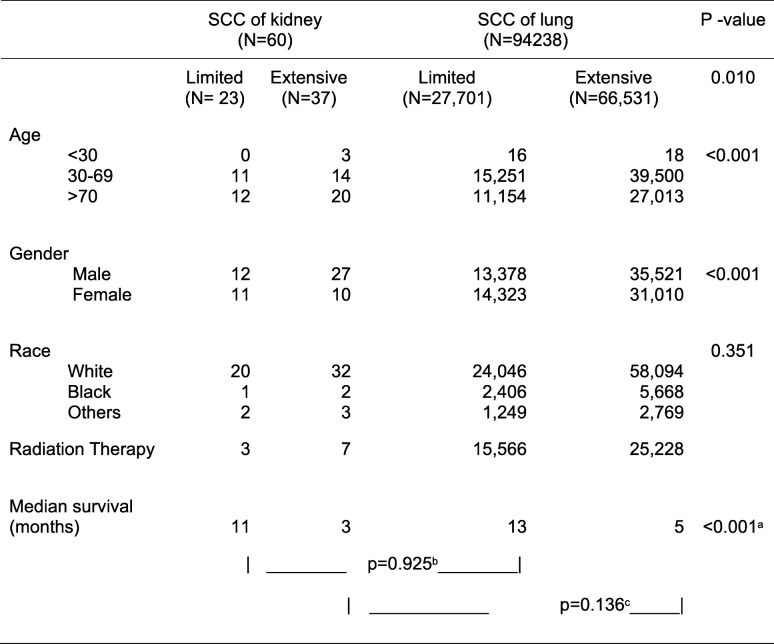
Comparison of cases between small cell carcinoma of kidney versus lung identified in Surveillance, Epidemiology, and End Results (1973–2013)

*SCC* small cell carcinoma. Log rank test: ^a^ overall, ^b^ limited, and ^c^ extensive stage small cell carcinoma of kidney versus lung

**Fig. 2:**
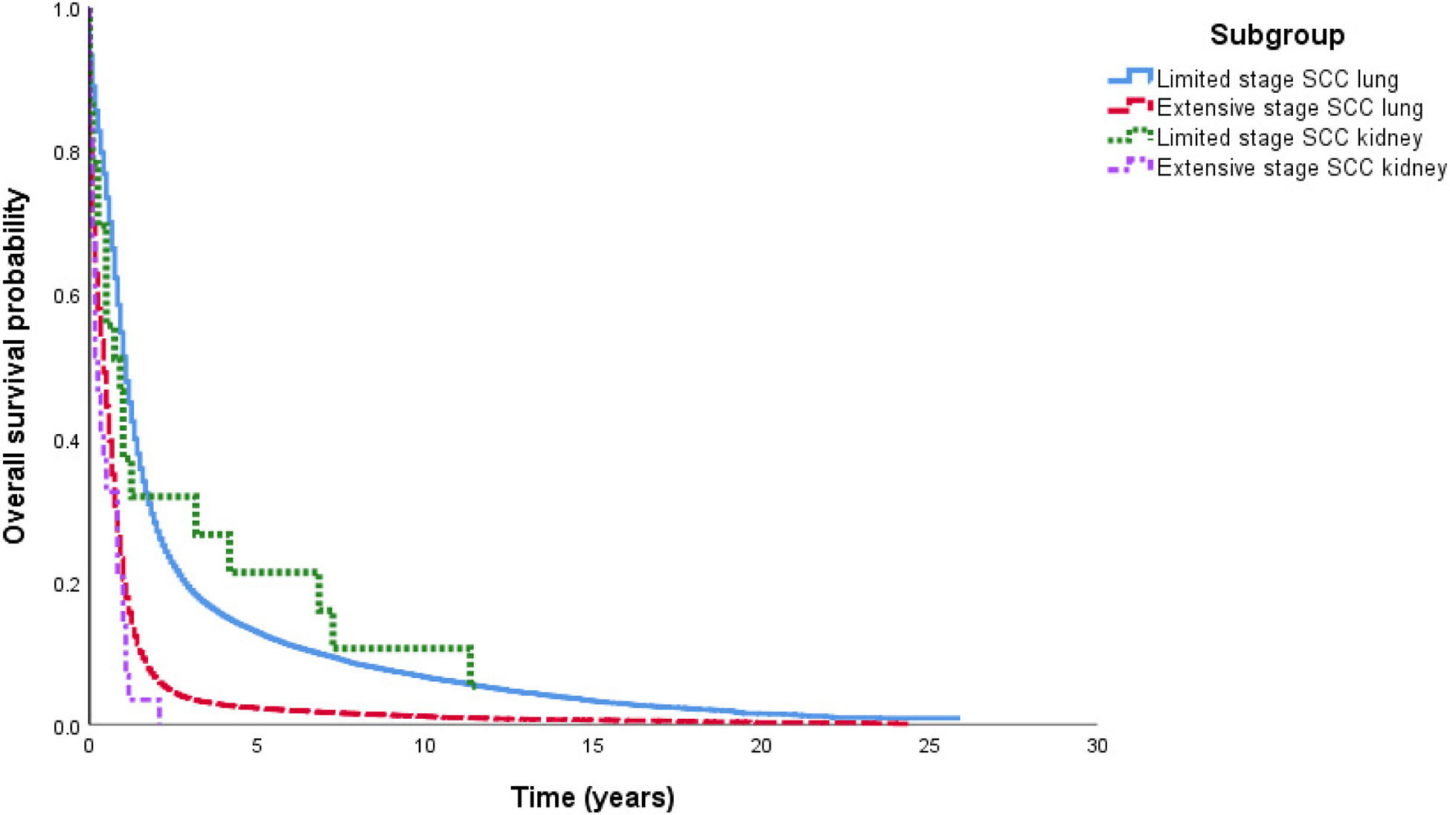
Overall survival by Kaplan–Meier analysis for small cell cancer of the kidney versus lung and limited versus extensive stage (log-rank test, overall, *p* < 0.001). *SCC* small cell carcinoma

Although not statistically different, patients with SCCL tended to live longer than patients with SCCK with a median survival of 13 versus 11 months (*p* = 0.925) in limited stage and 5 versus 3 months (*p* = 0.136) in extensive stage (Table 1). As expected, in SCCK, limited stage conferred much better outcome in survival than that of extensive stage: 11 versus 3 months, respectively (*p* = 0.003) (Fig. 2).

## Discussion

We present a case of small cell cancer of the kidney in a 69-year-old man. Our patient presented with hematuria and weight loss which is a similar presentation to other renal carcinomas. The case demonstrates a metastatic predilection to the central nervous system much like its lung counterpart. Chemotherapy was not pursued in part because of a delay in diagnosis and due to his poor functional status at time of consideration. SCCK is very aggressive and our patient died within 8 months of presentation. A review of the literature showed a similar level of aggressiveness. Our case adds the feature of SIADH to the limited case reports available thus far [2–4]. The development of SIADH has not been described in the literature of SCCK thus far. This suggests that the etiology of SIADH is a paraneoplastic feature of small cell cancers and not necessarily a feature based on the anatomic location in the lung. We add an additional case of SCCK and add an additional 60 cases based on the SEER registry to a growing body of literature regarding this rare cancer.

The primary objective of our study was to further characterize the clinical nature of SCCK by presenting a clinical scenario and using a population-based dataset from the SEER registry. Our SEER data directly compare SCCK and SCCL. Most cases of SCCK (62%) presented with an extensive stage similar to SCCL (70%). It typically occurred in white men in their seventh decade which is similar to SCCL. Our data suggest that the survival of SCCK is worse than SSCL, although not statistically significant due to our limited sample size. In comparing percentage survival of SCCK to SCCL we find that survival is much lower for SCCK. Patients with an extensive stage of SCCK had a median survival of 3 months versus 11 months in patients with limited stage of SCCK; this was worse than their counterparts in SCCL with a median survival of 5 and 13 months, respectively. One previous study of 45 patients demonstrated a median survival of 9.9 months among all cases of SCCK [7]. We have expanded this further by providing median survivals based on the extent of their disease. Further cases would be useful in drawing statistically significant conclusions.

There is currently no standard of care for SCCK and a lack of data to suggest benefit of chemotherapy, radiation therapy, surgical therapy, or any combination of treatment in SCCK. Given that SCCK and SCCL both present at limited and extensive stages at comparable percentages, we suspect that treatment differences may better account for the possible mortality differences, as opposed to early detection. Most cases of general renal cancers are managed surgically [3, 4, 8]. This bias may lead to underutilization of chemotherapy and/or radiation therapy in SCCK. Our case illustrates this phenomenon as chemotherapy was not offered until much later in the disease course as the initial management plan was surgical. Past case series have utilized nephrectomy alone, chemotherapy alone, or a combination of both; further case series are needed to establish a standard of care [3].

A small portion of the patients with SCCK in the SEER dataset received radiation therapy, 13.0% with limited stage and 18.9% with extensive stage, suggesting that the role of radiation therapy is still underutilized given the lack of a standard of care. It is also possible that due to the aggressive nature of SCCK, poor performance status may prevent the patients from being eligible to receive radiation therapy. Lastly, it is also unclear if these patients would benefit from radiation therapy in the first place. Radiation therapy may have a role in postoperative residual local disease or metastatic disease [9].

Much like SCCL, patients with SCCK may benefit from medical chemotherapy as well, as suggested by a systematic review examining 22 patients [3]. Another study, which examined 45 cases, suggested that early cisplatin-based chemotherapy offered a strong survival advantage [7]. Our case presented a patient who was not referred to a medical oncologist until later in his disease. By the time he was evaluated by his medical oncologist, he was not a candidate for chemotherapy due to his poor performance status. Perhaps, he would have had a better outcome if he had received chemotherapy at an earlier stage of his cancer. Furthermore, SEER data do not provide information regarding the use of chemotherapy. Further cases demonstrating the effects of chemotherapy on patients with SCCK would be informative.

There are several limitations to this study. SEER studies do not incorporate comorbidities or patient performance status which could influence overall mortality. Furthermore, SEER studies do not collect chemotherapy administration which could also influence overall mortality. Lastly, our sample size of SCCK only contained 60 patients. This made it difficult to draw statistically significant conclusions. However, this is the first population-based report of SCCK which is a strength of the current report.

## Conclusion

Despite its limitations, this is the first population-based analysis of SCCK using the SEER dataset. SCCK appears to be very aggressive with poor survival outcomes. Both SCCK and SCCL remain lethal diseases with poor prognosis and high mortality. Further investigations on the epidemiology of SCC may aid better understanding of its etiology leading to improvement in survival. Furthermore, more insight into treatment modalities is necessary. Our case demonstrated a delay in consideration of chemotherapy, which is probably due to most renal cancers being managed surgically.

## Abbreviations

CT: Computed tomography
SCC: Small cell carcinoma
SCCK: Small cell carcinoma of the kidney
SCCL: Small cell carcinoma of the lung
SEER: Surveillance, Epidemiology, and End Results
SIADH: Syndrome of inappropriate antidiuretic hormone secretion
